# Aerospace Mutagenesis Increases the Content of Characteristic Metabolites of Tea Tree Leaves and Enhances Taste Characteristics of Tea Leaves

**DOI:** 10.3390/foods13223538

**Published:** 2024-11-06

**Authors:** Jiaming Chen, Yangxin Luo, Qi Zhang, Yulin Wang, Tingting Wang, Miao Jia, Yankun Liao, Xiaoli Jia, Haibin Wang, Jianghua Ye

**Affiliations:** 1 College of Tea and Food, Wuyi University, Wuyishan 354300, China; jiamingchen2009@outlook.com (J.C.);; 2 College of Life Science, Longyan University, Longyan 364012, China

**Keywords:** aerospace mutagenesis, tea tree, metabolomics, characteristic metabolites, taste characteristics

## Abstract

Aerospace mutagenesis can alter the physiological metabolism and growth of tea trees and affect tea leaf quality. In this study, the effect of aerospace mutagenesis on the metabolite content and taste characteristics of Dahongpao fresh leaves and raw tea were analyzed. The results showed that aerospace mutagenesis had little effect on the composition and total amount of metabolites in fresh leaves and raw tea, but significantly changed the content of different metabolites. Aerospace mutagenesis improved the content of lipids, lignans and coumarins, and amino acids and derivatives, which in turn enhanced the aroma and fresh and brisk taste of fresh leaves. Aerospace mutagenesis increased the content of alkaloids, tannins, flavonoids, lignans and coumarins, amino acids and derivatives, and organic acids in raw tea, and enhanced the bitterness, mellowness, and fresh and brisk taste of raw tea. This study provides a basis for the development of aerospace mutagenesis Dahongpao tea products and the establishment of processing techniques.

## 1. Introduction

Aerospace mutagenesis is a special and novel technology for plant breeding. Plant seeds undergo significant changes in their heredity and gene regulation under the combined interference of multiple factors in unique environments, such as intense radiation, microgravity, high vacuum, and extreme temperatures, which do not occur under Earth’s gravity [1], which in turn facilitates rapid and efficient screening of high-quality germplasm resources [2]. China has made significant achievements in aerospace mutation breeding, and through aerospace breeding, more than 200 new varieties of plants have been developed and utilized in social production practices, which have made significant contributions to the development of agriculture [3,4]. For example, the Chinese Academy of Sciences (CAS) utilized aerospace mutagenesis to breed tomatoes and produced two high-yielding, disease-resistant, and high-quality varieties [5]. The new varieties of *Salvia miltiorrhiza* bred by Tasly Group using aerospace mutagenesis have significantly higher active ingredient content and three times the quality of other *S. miltiorrhiza* [6]. It is evident that aerospace mutagenesis has achieved remarkable results in the screening and breeding of excellent plant varieties.

Wuyishan is a premium tea-producing area in China. Dahongpao (*Camellia sinensis*) is one of the most important cultivated varieties in Wuyishan [7]. In 2011, Dahongpao seeds were placed onboard the unmanned spacecraft Shenzhou VIII for the first time to carry out space mutation, which lasted 16 days, 13 h, and 34 min. This is the first and, so far, the only time that Wuyi Rock Tea seeds have been obtained through aerospace mutagenesis. After the mutagenized Dahongpao tea tree seeds were germinated and planted, their tea tree leaf morphology already showed significant differences compared to the control at the seedling stage [8]. Jia et al. [9] analyzed the physiological characteristics and growth of tea tree, and found that, compared with the control, the surface of tea leaves after aerospace mutagenesis was smoother, the edge serrations were more compact and regular, and the photosynthetic capacity, resistance, and yield of tea trees were stronger. It is evident that the Dahongpao tea tree after aerospace mutagenesis had significant changes in its growth and yield. However, there are few reports on whether the quality of Dahongpao has also changed after aerospace mutagenesis in terms of quality.

Taste characteristics are one of the important indexes for evaluating tea quality, while the types of inclusions and their contents in tea leaves determine taste characteristics and affect tea quality [10,11]. Metabolomics technology can comprehensively and rapidly analyze metabolites in tea leaves and evaluate tea quality by metabolite content and taste characteristics [12,13]. Secondly, metabolomics technology can analyze and obtain key differential metabolites among different samples, which in turn facilitates rapid characterization of tea quality [14,15]. It was hypothesized that after aerospace mutagenesis, the type or content of metabolites in Dahongpao leaves changed significantly, which in turn affected the taste characteristics of fresh leaves or raw tea; especially, the type and content of characteristic metabolites in the leaves were closely related to taste characteristics. Based on this, Dahongpao fresh leaves were collected from aerospace-mutagenized and non-aerospace-mutagenized Dahongpao tea tree in this study. Metabolomics techniques were used to analyze the types of metabolites and their contents in tea tree fresh leaves and raw tea. We further screened and analyzed the characteristic metabolites of the two samples and analyzed their taste characteristics to clarify the effect of aerospace mutagenesis on the quality of Dahongpao tea, with a view to laying a foundation for the development of aerospace mutagenized Dahongpao tea products and the establishment of processing technology.

## 2. Materials and Methods

### 2.1. Materials

The experimental reagents were mainly methanol, acetonitrile, and formic acid, which were chromatographically pure and purchased from German Merck Shanghai branch (Shanghai, China).

Tea samples for this study were obtained from the Tea Tree Aerospace Breeding Experimental Base, Wuyishan, Fujian, China (117°59′47.7″ E, 27°44′8.4″ N). The research variety selected was Dahongpao (*C. sinensis*) tea tree, and the age of aerospace-mutagenized and non-aerospace-mutagenized tea trees at the time of sampling was 11 years. The main process of aerospace mutagenesis of tea tree seeds was, at 5:58 on 1 November 2011, the same batch of tea tree seeds mixed well, part of the seeds with the “Shenzhou VIII” unmanned spacecraft launched for aerospace mutagenesis, and part of the seeds remained on the ground as the control.

Two days after the lift-off of Shenzhou VIII, which carried tea tree seeds, it docked with the Tiangong I target vehicle and operated as a single unit for 12 days. And at 18:30 on 16 November 2011, the assemblage separated. The capsule successfully returned to Earth at 19:32 on 17 November 2011, with a total of 16 days, 13 h, and 34 min of space mutation time. In April 2012, the mutagenized seeds of the tea tree were germinated and then planted at the same time as the control, on the same plot, and the tea trees were uniformly managed in the same way.

In May 2023, Dahongpao fresh leaves without aerospace mutagenesis (CK) and with aerospace mutagenesis (TM) were picked in three replicates of 50 kg each. The picking standard for tea tree fresh leaves was one bud and three leaves. After each replicate sample was thoroughly mixed, 2 kg of each replicate were used for leaf metabolite extraction and identification. The remaining 48 kg of fresh leaves were taken for the preliminary processing to make raw tea. The method of preliminary processing of tea leaves was in accordance with the standard preliminary processing method of Wuyi rock tea, with specific reference to the method of Ye et al. [16]. Briefly, fresh tea leaves were withered for 30 min under 57,000 lux light, shaken for 450 min at room temperature of 27 °C and humidity of 70%, killed for 8 min at 260 °C, kneaded for 8 min, and dried for 50 min at 110 °C. Then, the raw tea could be obtained. Raw tea processed from CK (FCK) and raw tea processed from TM (FTM) were collected in 2 kg of three independent replicates per sample. The collected raw tea was used for metabolite extraction and identification.

### 2.2. Determination of Tea Metabolites

Extraction and determination of tea metabolites followed the method of Zhou et al. [17]. The tea samples were freeze-dried by vacuum freeze-dryer (Scientz-100F, Zhejiang, China) and then ground to powder. Then, 50 mg powder was added to 1.2 mL methanol solution (70%) before vortex oscillation for 30 s. The mixture was vortexed and shaken for 30 s at 30 min intervals 6 times. After the above mixture was then centrifuged at 12,000 rpm for 3 min, it was passed through a 0.22 μm filter membrane for ultra-performance liquid chromatography/tandem mass spectrometry (UPLC-MS/MS) analysis.

The above filtrates were determined by UPLC-MS/MS (UPLC: Shimadzu, Nexera X2, Kyoto, Japan; MS/MS: Applied Biosystems 4500 QTRAP, Framingham, MA, USA). The UPLC column utilized was Agilent SB-C18 (1.8 µm, 2.1 mm × 100 mm, Santa Clara. CA, USA). For the mobile phase, ultrapure water (containing 0.1% formic acid) served as phase A, and acetonitrile (also containing 0.1% formic acid) served as phase B. The UPLC assay was performed using a gradient elution mode as follows: the phase B ratio was 5% at 0.00 min, the phase B ratio increased linearly to 95% within 9.00 min and was maintained at 95% for 1 min (10.00–11.10 min), the phase B ratio was reduced to 5% and equilibrated at 5% up to the 14th min. The flow rate was set to 0.35 mL/min, and the column temperature was kept at 40 °C. Additionally, an injection volume of 2 μL was employed. MS/MS experiments were carried out under optimized conditions, specifically an electrospray ionization temperature of 500 °C. The positive ion mode was set to 5500 V and the negative mode was adjusted to −4500 V in the ion spray voltage settings. The ion source gas I was set to 50 psi, the ion source gas II was set to 60 psi, and the captain gas was set to 25 psi. The collision-activated dissociation was set to high. Triple-series quadrupole mass spectrometry was employed and operated in multiple reaction monitoring mode, with the collision gas set to medium. The declustering potential and collision energy were refined and optimized to obtain the appropriate declustering potential and collision energy of each multiple reaction monitoring ion. Then, based on the metabolites eluted within each period, each period was set to monitor a specific set of multiple reaction monitoring ion pairs.

The qualitative determination of metabolites was briefly described by first processing the mass spectrometry data using Analyst (1.6.3) software to obtain retention times and ion current intensities of metabolites, and then comparing them with the NIST20 mass spectrometry database to qualify metabolites. Quantitative determination of metabolites was briefly described as follows: The triple quadrupole was used to screen for characteristic ions of each substance. The detector was used to obtain the signal intensities of these characteristic ions. MultiQuant (3.0) software was used to integrate and correct the peaks, and the peak area of each peak represented the relative content of the corresponding substance [18].

### 2.3. Statistical Analysis

Excel 2020 was used for preliminary statistical analysis of raw metabolite data obtained, including metabolite classification, content calculation, etc. Analysis of paired Student’s *t*-tests and variance (ANOVA) were used uniformly to analyze differences in data between different samples. Graphs were produced using Rstudio software (v 4.2.3), with box plots produced using the R package gghalves 0.1.4, principal component plots produced using the R package ggbiplot 0.55, bubble heat maps produced using the R package ggplot2 3.5.1, and volcano plots produced using the R package ggplot2 3.5.0. The R packages used for orthogonal partial least squares discrimination analysis (OPLS-DA) model construction for CK and TM, and FCK and FTM were all ropls and mixOmics. The R package used for bubble feature map production was ggplot2 3.4.4. The R packages used for taste wheel production were ComplexHeatmap 2.16.0, circlize v0.4.15, and RColorBrewer v1.1.3.

## 3. Results and Discussion

### 3.1. Quantity and Type Analysis of Metabolites in Dahongpao Fresh Leaves and Raw Tea

**Figure 1:**
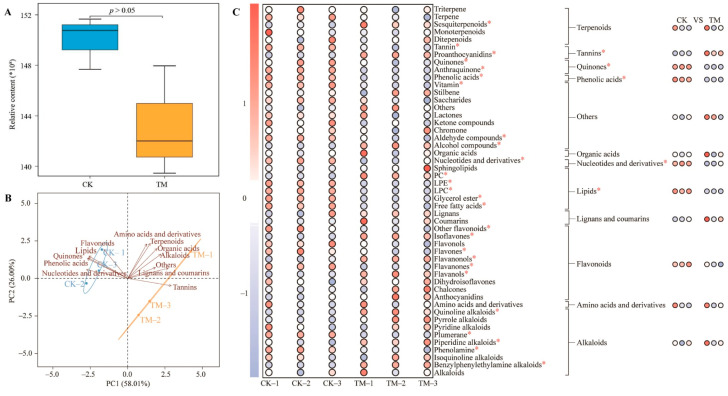
Analysis of metabolite content and types in fresh leaves of Dahongpao tea tree. Note: CK: Dahongpao fresh leaves without aerospace mutagenesis; TM: Dahongpao fresh leaves after aerospace mutagenesis; (**A**) analysis of total amount of metabolites in CK and TM; (**B**) PCA analysis after categorization of metabolites in CK and TM; (**C**) content analysis of different categories of metabolites in CK and TM; * denotes that the difference between two samples reaches the *p* < 0.05 level.

Aerospace mutation breeding is an efficient and fast breeding method [19]. Plants after aerospace mutagenesis experience significant changes in growth, physiology, and metabolism, which in turn lead to significant differences in metabolite composition and content [20]. Xia et al. [3] used GC-MS to find the changes in metabolite composition of andrographis paniculata after aerospace mutagenesis and indicated that aerospace mutagenesis effectively improved the content of different metabolites and the quality of andrographis paniculata. In the present study, metabolomics was utilized to analyze the effects of spaceflight mutagenesis on metabolites in fresh leaves and raw tea of Dahongpao (Tables S1 and S2). Among them, metabolite analysis of fresh leaves showed (Figure 1A) that a total of 2526 metabolites were detected, and non-aerospace-mutagenized Dahongpao fresh leaves (CK) and aerospace-mutagenized Dahongpao fresh leaves (TM) did not differ significantly in the total number of metabolites (*p* > 0.05). The obtained 2526 metabolites were further classified and analyzed for their contents, and the results showed (Table S1) that the detected metabolites could be classified into 12 groups according to the first-level classification method, and the top 5 metabolites with the highest number were flavonoids (23.40%), phenolic acids (17.14%), alkaloids (8.79%), amino acids and derivatives (7.76%), and lipids (7.60%). PCA analysis with metabolites detected in CK and TM and their contents showed (Figure 1B) that the two principal components could distinguish CK and TM effectively with an overall contribution of 84.01%. Further analysis showed that there were five categories of metabolites associated with CK, namely, quinones, phenolic acids, nucleotides and derivatives, phenolic acids, lipids, flavonoids, and seven categories associated with TM, namely, terpenoids, organic acids, lignans and coumarins, amino acids and derivatives, tannins, alkaloids, and others. It is evident that the composition and total number of metabolites in fresh leaves did not change significantly after aerospace mutagenesis, but different metabolites contained significant differences in their content.

Therefore, further analysis of metabolite content in this study revealed (Figure 1C) that 2526 metabolites could be categorized into 49 groups according to the secondary classification method, of which 10 groups were significantly greater in TM than CK, 16 groups were significantly less in TM than CK, and 23 groups were insignificantly different between TM and CK, whereas, among the 12 groups of metabolites obtained according to the first-level classification method, the content of 1 group (tannins) was significantly higher than that of CK, the content of 4 groups (quinones, phenolic acids, nucleotides and derivatives, lipids) was significantly lower than that of CK, and the content of 7 groups did not differ significantly between TM and CK. It is evident that the metabolite content of fresh leaves changed significantly after aerospace mutagenesis.

**Figure 2:**
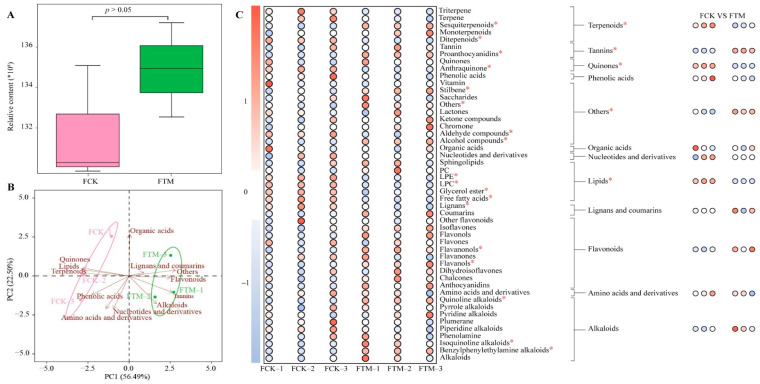
Analysis of metabolite content and types in raw tea of Dahongpao. Note: FCK: raw tea processed from Dahongpao fresh leaves without aerospace mutagenesis; FTM: raw tea processed from Dahongpao fresh leaves after aerospace mutagenesis; (**A**) analysis of total amount of metabolites in FCK and FTM; (**B**) PCA analysis after categorization of metabolites in FCK and FTM; (**C**) content analysis of different groups of metabolites in FCK and FTM; * denotes that the difference between two samples reaches the *p* < 0.05 level.

Regarding raw tea, the present study also analyzed the composition and content of metabolites, and the results revealed (Figure 2A) that a total of 2529 metabolites were obtained from raw tea. In terms of total number of metabolites, the difference between raw tea without aerospace mutagenesis (FCK) and raw tea after aerospace mutagenesis (FTM) was not significant (*p* > 0.05). The metabolites obtained were analyzed according to the first-level classification method and found (Table S2) to be classified into 12 groups, of which the top 5 groups with the greatest number were flavonoids (23.41%), phenolic acids (17.16%), alkaloids (8.82%), amino acids and derivatives (7.79%), and lipids (7.59%). PCA analysis showed (Figure 2B) that the two principal components could effectively differentiate FCK and FTM with a total contribution of 78.99%. Of these, the six groups associated with FCK were quinones, lipids, terpenoids, amino acids and derivatives, phenolic acids, and nucleotides and derivatives, while the six groups associated with FTM were tannins, organic acids, lignans and coumarins, flavonoids, alkaloids, and others. As can be seen, FCK and FTM remained similar in metabolite composition, but there were significant differences in the content of each metabolite. Accordingly, the classification of metabolites and the analysis of their content showed (Figure 2C) that 2529 metabolites could be classified into 49 groups according to the second-level classification method, in which the content of 10 groups was significantly higher in FTM than in FCK, the content of 8 groups was significantly lower in FTM than in FCK, and the content of 31 groups was insignificant between FTM and FCK. In contrast, metabolites were analyzed according to the first-level classification method into 12 groups, in which the content of tannins and others was significantly higher in FTM than in FCK, while the content of terpenoids, quinones, and lipids was significantly less in FTM than in FCK. It is evident that after aerospace mutagenesis, the metabolites in fresh leaves and raw tea are more similar in composition, and there is no significant difference in the total number of metabolites, but the content of each metabolite has changed significantly.

To sum up, aerospace mutagenesis can change the content of different metabolites in fresh leaves, which in turn leads to significant differences in the content of different metabolites in raw tea.

### 3.2. Screening for Characteristic Metabolites in Dahongpao Fresh Leaves and Raw Tea

**Figure 3:**
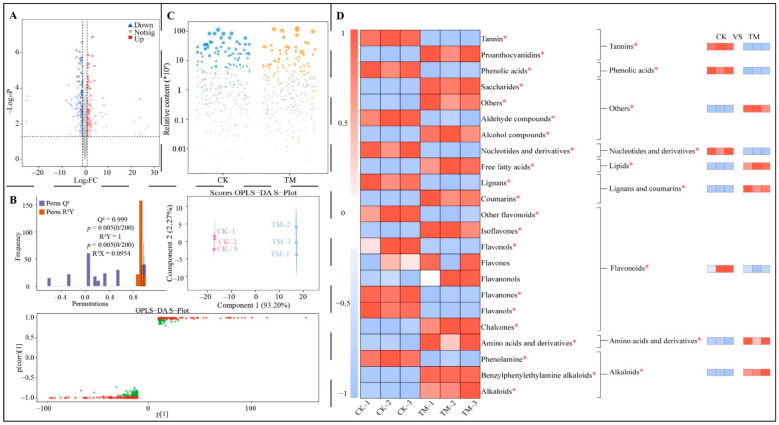
Screening and content analysis of characteristic metabolites in fresh leaves of Dahongpao tea tree. Note: CK: Dahongpao fresh leaves without aerospace mutagenesis; TM: Dahongpao fresh leaves after aerospace mutagenesis; (**A**) screening of differential metabolites in CK and TM by volcano map; (**B**) OPLS-DA model construction of CK and TM to screen key differential metabolites; (**C**) screening of characteristic metabolites in CK and TM by bubble feature map; (**D**) classification and content analysis of characteristic metabolites; * denotes that the difference between two samples reached the *p* < 0.05 level.

After the above analysis, the present study further analyzed characteristic metabolites that significantly altered in fresh leaves and raw tea after aerospace mutagenesis. The OPLS-DA model is an effective way to obtain key metabolites that can distinguish different samples [21]. By constructing the model, inter- and intragroup differences of different samples can be analyzed, while variable importance projection (VIP) values of different metabolites in distinguishing different samples can be obtained, and then key metabolites with VIP greater than 1 can be obtained [22]. However, after the OPLS-DA model is constructed, the model needs to be evaluated, and the model can be used for analysis if the model’s goodness-of-fit and predictability are both met [23]. Accordingly, in this study, the volcano diagram was first used for the preliminary screening of metabolites in fresh leaves, and then the OPLS-DA model was constructed to screen the key metabolites. The results of volcano diagram analysis showed (Figure 3A) that 148 metabolite contents in TM increased significantly, 230 metabolite contents decreased significantly, and 2148 metabolite contents were not significantly different from CK. The OPLS-DA model of CK and TM was further constructed based on 378 metabolites and their contents that were screened for significant differences in the volcano diagram, and it was found (Figure 3B) that the R^2^Y value of the fit of the constructed model reached a significant level (*p* < 0.005), as well as the Q^2^ value of predictability (*p* < 0.005). It can be seen that the constructed model meets the requirements and can be used for analysis. From the score plot of the model, the model can effectively differentiate between CK and TM, with a 93.20% between-group difference and less than 2.27% within-group difference. It is evident that the OPLS-DA model of CK and TM can effectively distinguish between the two samples. Based on this, VIP values of different metabolites distinguishing CK and TM were derived from the S-plot of the model, and 253 key metabolites with VIP > 1 were screened, of which the content of 105 metabolites was significantly larger in TM compared to CK and the content of 148 metabolites was significantly smaller in TM. A bubble feature map was further used to screen for characteristic metabolites distinguishing CK and TM, and it was found (Figure 3C) that 87 characteristic metabolites were obtained, of which the content of 35 metabolites was significantly larger in TM compared to CK, and the content of 52 metabolites was significantly smaller in TM. The results of characteristic metabolite classification and content analysis of each group showed (Figure 3D) that 87 metabolites could be classified into 23 groups according to the second-level classification method, of which 11 groups were significantly higher in TM compared to CK, 10 groups were significantly less in TM, and the difference of 2 groups between CK and TM was not significant. In addition, according to the first-level classification method, the 87 metabolites could be classified into 9 groups, in which the content of lipids, alkaloids, lignans and coumarins, amino acids and derivatives, and others was significantly higher in TM than in CK, and the content of tannins, flavonoids, phenolic acids and nucleotides, and derivatives was significantly smaller in TM.

It is evident that the metabolite content in fresh leaves has changed significantly after aerospace mutagenesis, especially characteristic metabolites, and this change may affect the content of each metabolite in their raw tea.

**Figure 4:**
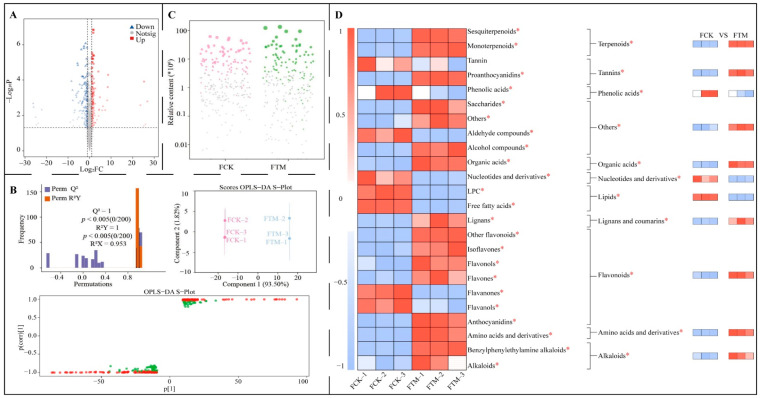
Screening and content analysis of characteristic metabolites in raw tea of Dahongpao tea tree. Note: FCK: raw tea processed from Dahongpao fresh leaves without aerospace mutagenesis; FTM: raw tea processed from Dahongpao fresh leaves after aerospace mutagenesis; (**A**) screening of differential metabolites in FCK and FTM by volcano diagram; (**B**) OPLS-DA model construction of FCK and FTM to screen key differential metabolites; (**C**) screening of characteristic metabolites in FCK and FTM by bubble feature plot; (**D**) classification and content analysis of characteristic metabolites; * indicates that the difference between two samples reached the *p* < 0.05 level.

However, it is worth noting that the transformation of substances within tea leaves during processing ultimately leads to the formation of the distinctive taste that makes tea unique. This flavor development is driven by the content of different metabolites initially present in fresh leaves, which act as the substrate for the chemical reactions that occur during processing [24,25]. The higher the metabolite content and the richer the type of metabolites in fresh leaves, the more conducive they are to processing, resulting in the accumulation of different types of metabolites in raw tea and the formation of a unique taste of made tea [26,27]. Hereby, the present study further analyzed the characteristic metabolites in raw tea after aerospace mutagenesis, and the volcano diagram analysis showed (Figure 4A) that the content of 41 metabolites increased significantly, the content of 182 metabolites decreased significantly, and the difference in the content of 2206 metabolites was not significant in FTM compared to FCK. The OPLS-DA model based on FCK and FTM was further constructed, and showed (Figure 4B) that the R2Y value of the model fit and the Q2 value of predictability reached significant levels (*p* < 0.005). The model was effective in distinguishing FCK from FTM, with a difference of 93.50% between groups and less than 1.82% within groups. The OPLS-DA model of FCK and FTM meets the requirements and can effectively distinguish the two samples. Secondly, the VIP values of different metabolites were derived from the S-plot of the model, and 229 key metabolites with VIP > 1 were obtained, of which the content of 105 metabolites was significantly higher in FTM compared with FCK, and the content of 124 metabolites was significantly less in FTM. The bubble feature map was further used to screen for characteristic metabolites distinguishing FCK and FTM, and showed (Figure 4C) that 97 characteristic metabolites were obtained, of which 49 metabolites were significantly higher in FTM compared to FCK, and 48 metabolites were significantly lower in FTM. The classification of the characteristic metabolites and the content of each group classified showed (Figure 4D) that 97 metabolites could be classified into 24 groups according to the second-level classification, 16 groups of FTM had a significantly greater content than FCK, 7 groups of FTM had a significantly smaller content than FCK, and 1 group had a nonsignificant difference between FCK and FTM. However, according to the first-level classification, the 97 metabolites were categorized into 11 groups, in which the content of terpenoids, organic acids, lignans and coumarins, tannins, flavonoids, amino acids and derivatives, alkaloids, and others was significantly greater in FTM compared to FCK, while the content of phenolic acids, nucleotides and derivatives, and lipids was significantly less in FTM. It is evident that the metabolite content between FTM and FCK differed significantly. It was also found in this study that some metabolites with higher contents in TM compared to CK, e.g., alkaloids, amino acids and derivatives, and lignans and coumarins, remained at higher levels after processing, and FTM was significantly greater than FCK. Conversely, some metabolites with lower levels in TM, e.g., phenolic acids, nucleotides, and derivatives, remained low after processing, and FTM was significantly smaller than FCK. It is evident that there is a close relationship between the metabolite content of fresh leaves and the metabolite content of raw tea, especially characteristic metabolites.

### 3.3. Taste Characteristics and Variation Analysis of Characteristic Metabolites in Dahongpao Fresh Leaves and Raw Tea

**Figure 5:**
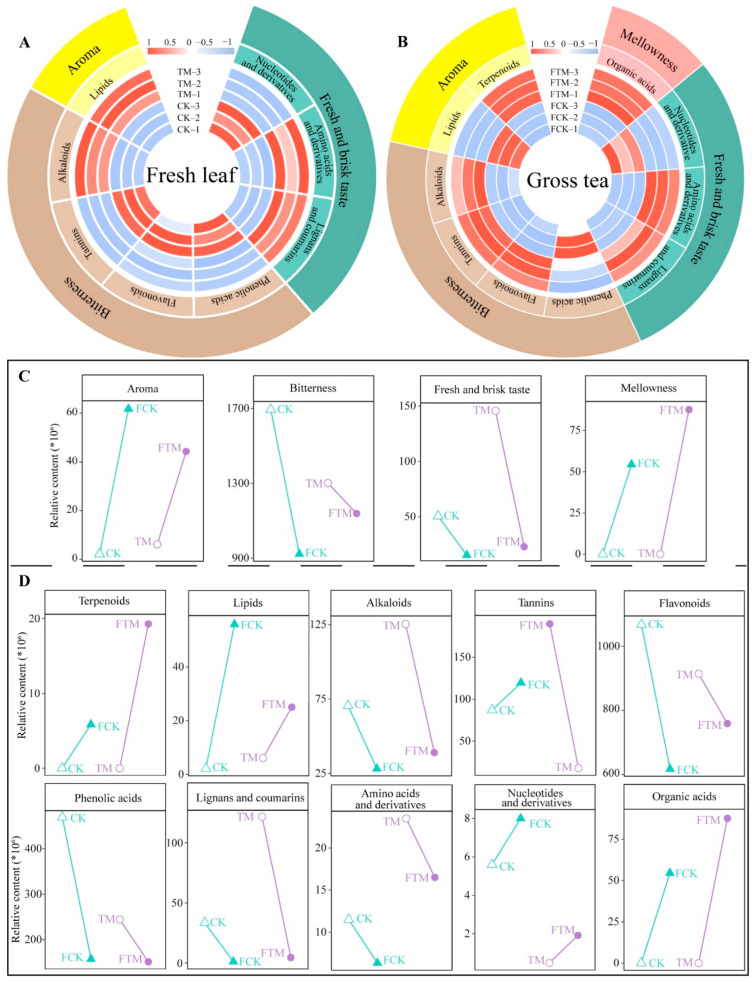
Taste and content analysis of characteristic metabolites in fresh leaves and raw tea of Dahongpao tea tree. Note: CK: Dahongpao fresh leaves without aerospace mutagenesis; TM: Dahongpao fresh leaves after aerospace mutagenesis; FCK: raw tea processed from Dahongpao fresh leaves without aerospace mutagenesis; FTM: raw tea processed from Dahongpao fresh leaves after aerospace mutagenesis; (**A**) taste and intensity analysis of the characteristic metabolites in CK and TM; (**B**) taste and intensity analysis of the characteristic metabolites in FCK and FTM; (**C**) changes in the content of different metabolites in fresh leaves and raw tea; (**D**) changes in the taste of the characteristic metabolites in fresh leaves and raw tea.

The evaluation of tea quality is significantly influenced by taste, and the formation of tea taste characteristics is highly correlated with the types and proportions of metabolites present in tea [28]. It has been reported that nucleotides and derivatives, lignans and coumarins, amino acids, and derivatives are found in tea and the main taste characteristic is fresh and brisk taste [29,30,31]. The taste characteristics of phenolic acids, flavonoids, tannins, and alkaloids mainly exhibit bitterness [32,33,34]. The taste characteristics of terpenoids and lipids mainly present aroma [35,36], whereas the taste characteristics of organic acids present a mellowness [37]. Accordingly, the present study analyzed taste characteristics based on the abovementioned characteristic metabolites obtained from fresh leaves and raw tea, and found that the characteristic metabolites of CK and TM exhibited mainly fresh and brisk taste, bitterness, and aroma (Figure 5A). However, the characteristic metabolites of FCK and FTM exhibited mainly mellowness, bitterness, fresh and brisk taste, and aroma (Figure 5B). Further analysis of the taste characteristics of fresh leaves and raw tea revealed (Figure 5C) that, from the taste characteristics of fresh leaves, aroma, and fresh and brisk taste of TM were higher than that of CK, while its bitterness was lower than that of CK; from the taste characteristics of raw tea, bitterness, mellowness, and fresh and brisk taste of FTM were greater than those of FCK, while its aroma was lower than that of FCK. Analysis from the point of view of taste characteristics revealed that the processing of fresh tea leaves into raw tea resulted in a significant enhancement of both aroma and mellowness, while bitterness and fresh and brisk taste were significantly reduced. It is evident that processing increases the intensity of aroma and mellowness of tea and reduces bitterness and fresh and brisk taste. Compared to CK, TM has a stronger aroma and fresh and brisk taste, while FTM has a stronger bitterness, fresh and brisk taste, and mellowness compared to FCK.

In addition, further analysis of metabolites with different taste characteristics in fresh leaves and raw tea in the present study revealed (Figure 5D) that from the perspective of fresh leaves, the content of lipids, alkaloids, lignans and coumarins, amino acids, and derivatives was greater in TM than in CK, whereas the content of tannins, flavonoids, phenolic acids, nucleotides, and derivatives was lower than CK. From the point of raw tea, FTM had a higher content of terpenoids, alkaloids, tannins, flavonoids, lignans and coumarins, organic acids, and amino acids and derivatives than FCK, while its content of lipids, nucleotides, and derivatives were lower than FCK. Analysis of metabolite content revealed that the content of terpenoids, lipids, nucleotides and derivatives, tannins, and organic acids increased significantly from fresh leaves to raw tea, while the content of alkaloids, flavonoids, lignans and coumarins, phenolic acids, amino acids, and derivatives decreased significantly. It can be seen that TM has a higher content of lipids, amino acids, lignans and coumarins, and derivatives and its aroma and fresh and brisk taste is stronger as compared to CK, whereas FTM has higher content of alkaloids, tannins, flavonoids, lignans and coumarins, organic acids, amino acids, and derivatives, and its bitterness, fresh and brisk taste, and mellowness were stronger than FCK.

## 4. Conclusions

**Figure 6:**
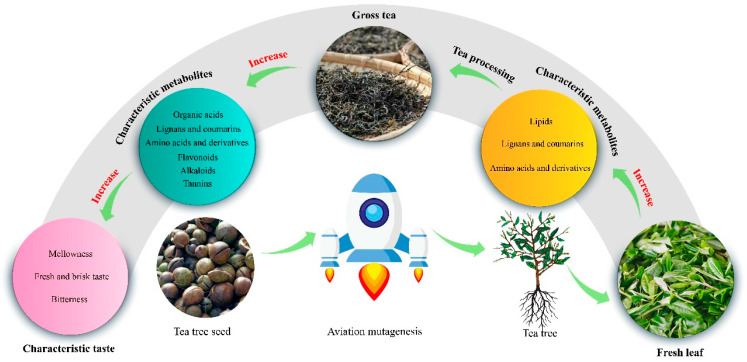
Mechanism analysis of the influence of aerospace mutagenesis on tea taste characteristics.

In the present study, the effect of aerospace mutagenesis on metabolite contents and taste characteristics in Dahongpao fresh leaves and raw tea was analyzed, and it was found (Figure 6) that the metabolites in fresh leaves and raw tea after aerospace mutagenesis were more similar in composition, and there was no significant difference in the total number of metabolites, but the content of different metabolites changed significantly. There were 87 characteristic metabolites distinguishing CK from TM and 97 characteristic metabolites distinguishing FCK from FTM. Taste characteristics analysis showed that taste characteristics of the characteristic metabolites in CK and TM were mainly fresh and brisk taste, bitterness, and aroma, whereas taste characteristics of characteristic metabolites in FCK and FTM were mainly fresh and brisk taste, bitterness, mellowness, and aroma. Aroma and fresh and brisk taste in TM were higher than CK, while bitterness was lower than CK. Fresh and brisk taste, bitterness, and mellowness were higher in FTM than in FCK, while aroma was lower than FCK. Processing increased the strength of aroma and mellowness and decreased bitterness and fresh and brisk taste of the tea. Aroma and fresh and brisk taste were stronger in TM compared to CK, while fresh and brisk taste, bitterness, and mellowness were stronger in FTM compared to FCK. In conclusion, TM had higher content of lipids, amino acids and derivatives, and lignans and coumarins, and its aroma and fresh and brisk taste was stronger compared to CK, while FTM had a higher content of alkaloids, tannins, amino acids and derivatives, flavonoids, lignans and coumarins, amino acids and derivatives, and organic acids, and its bitterness, fresh and brisk taste, and mellowness were stronger than FCK. It is evident that aerospace mutagenesis effectively altered the content of characteristic metabolites in tea leaves, which in turn altered their taste characteristics. This study provides a basis for the development of Dahongpao tea products by aerospace mutagenesis and the establishment of processing technology.

## Data Availability

The original contributions presented in the study are included in the article/Supplementary Materials, further inquiries can be directed to the corresponding authors.

## Supplementary Materials

The following supporting information can be downloaded at: https://www.mdpi.com/article/10.3390/foods13223538/s1, Table S1: Raw data of metabolites in fresh leaves of Dahongpao; Table S2: Raw data of metabolites in raw tea of Dahongpao.
